# Effectiveness of inspiratory muscle training associated with a cardiac rehabilitation program on sympathetic activity and functional capacity in patients with heart failure: a study protocol for a randomized controlled trial

**DOI:** 10.1186/s13063-020-04363-6

**Published:** 2020-06-12

**Authors:** Jéssica Costa Leite, Daniella Cunha Brandão, Simone Cristina Soares Brandão, Helen Kerlen Bastos Fuzari, Tainá Maria Vidal, Jasiel Frutuoso, Maria Inês Remígio, Bruna Thays Santana de Araújo, Shirley Lima Campos, Armele Dornelas de Andrade

**Affiliations:** 1 Department of Physiotherapy, Universidade Federal do Rio Grande do Norte, Av. Senador Salgado Filho, s/n, Natal, 59078-970 Rio Grande do Norte Brazil; 2 Department of Physiotherapy, Universidade Federal de Pernambuco, Av. Jornalista Aníbal Fernandes, s / n, Recife, Pernambuco 50740-560 Brazil; 3 Nuclear Medicine and Cardiology Services, Hospital das Clínicas de Pernambuco, Universidade Federal de Pernambuco, Av. Prof. Moraes Rego, 1235, Recife, 50670-901 Pernambuco Brazil; 4 Department of Clinical Semiology of the Medicine Faculty, Universidade Federal de Pernambuco, Av. Jornalista Aníbal Fernandes, s / n, Cidade Universitária, Recife, 50740-560 Pernambuco Brazil

**Keywords:** Heart failure, Sympathetic hyperactivation, Functional capacity, Cardiac rehabilitation, Inspiratory muscle training

## Abstract

**Background:**

Individuals affected by heart failure (HF) may present fatigue, dyspnea, respiratory muscle weakness, and sympathetic activity hyperstimulation of the myocardium, among other symptoms. Conducting cardiac rehabilitation (CR) programs can be associated with inspiratory muscle training. The aim of this study was to evaluate the efficacy of inspiratory muscular training (IMT) associated with a CR program on modulating myocardial sympathetic activity and maximal functional capacity, submaximal functional capacity, thickness, and mobility of the diaphragm muscle in patients with HF.

**Methods:**

We will conduct a clinical, controlled, randomized, double-blind trial that will include sedentary men and women who are 21–60 years old and who have diagnosed systolic HF and a left ventricular ejection fraction of less than 45%. Participants will be randomly assigned to one of two groups: experimental and control. The control group will follow the conventional CR protocol, and the experimental group will follow the conventional CR protocol associated with IMT 7 days a week. The two proposed exercise protocols will have a frequency of three times a week for a period of 12 weeks. The sympathetic innervation of the cardiac muscle, the maximum and submaximal functional capacity, diaphragm mobility and thickness, and the quality of life of the participants will be evaluated before and after the intervention protocol.

**Discussion:**

This clinical trial will be the first study to investigate the additional effects of IMT on CR in sympathetic hyperstimulation in the myocardium. The results of this study will contribute to developing therapeutic strategies collaborating to elucidate whether the association of IMT with CR can induce clinical benefits for patients with HF.

**Trial registration:**

ClinicalTrials.gov identifier: NCT02600000. Registered November 9, 2015. Retrospectively registered.

## Background

Heart failure (HF) is a cardiovascular syndrome with an impact on public health because of the high morbimortality rates with which it is associated. The main symptoms of HF are dyspnea, fatigue, and reduced functional capacity [1, 2]. In addition, autonomic nervous system (ANS) imbalance and aggravation of symptoms may occur as a consequence of adaptation to HF [1].

Given the importance of the ANS in patients with cardiopathy, cardiac sympathetic activity and innervation should be evaluated, and scintigraphy with metaiodobenzylguanidine labeled with iodine 123 (I^123^ MIBG) is an effective and indicated method for this type of analysis. An image obtained 15 minutes after administration of the radiopharmaceutical represents the integrity of the presynaptic nerve terminals and the density of the beta-adrenergic receptors. Four hours later, presynaptic neuronal uptake contributes to late imaging, combining information on neural function, including uptake, release, and storage of noradrenaline in the presynaptic vesicles. In addition to these, the washout rate (WR) is evaluated, constituting a parameter that shows the degree of sympathetic activity, and its increase reflects the overflow of noradrenaline into the bloodstream [3–6].

Changes in sympathetic activity also appear to be related to reduced functional capacity in these individuals as well as other factors such as the restrictive pulmonary ventilatory pattern, aggravated by the progressive increase of the cardiac area, respiratory muscle dysfunction, and peripheral musculature. However, these findings are still the subject of many investigations in order to relate respiratory dysfunctions to the reduction in exercise capacity in these individuals [6, 7].

Within this context, the inclusion of inspiratory muscular training (IMT) within cardiac rehabilitation (CR) programs has been increasingly disseminated as a good strategy to improve the clinical-functional findings in patients with HF associated with an increase in exercise performance. IMT can reduce symptoms such as dyspnea and inspiratory muscle fatigue through its effects on cardiovascular and respiratory systems yet is still not as widely used; this is perhaps because few data on its effect on the functional capacity of individuals with HF are available [8, 9].

A systematic review analyzed the effect of IMT on patients with HF, and it confirmed—among other results—improvement in maximal oxygen consumption (VO_2_) through a 12-week program. This improvement can be attributed to the delay in the development of diaphragmatic fatigue in patients with HF, leading to a reduction in the activation of additional respiratory muscles, increasing ventilatory efficiency, and reducing the blood flow required by the respiratory muscles during exercise, improving systemic vasodilation and perfusion of peripheral muscles, thereby reducing sympathetic activation [10]. However, of all the studies selected in the review, only one associated IMT with another technique (aerobic exercise) [11], and none of the studies performed a direct evaluation of myocardial sympathetic activity through radiopharmaceutical scintigraphy; it was evaluated only indirectly through spirometric parameters.

Considering the above, we hypothesized that an association of IMT with CR has modulating action on cardiac autonomic response and peripheral sympathetic activity in patients with HF and has effects on improving the maximum and submaximal functional capacity when compared with CR without IMT. It is also capable of increasing the thickness and mobility of the diaphragm muscle in these patients. Thus, the aim of this study was to evaluate the efficacy of inspiratory muscle training associated with a CR program on modulating myocardial sympathetic activity and maximal functional capacity (primary outcome), submaximal functional capacity (secondary outcome), and diaphragm muscle thickness and mobility (secondary outcome) in patients with HF.

## Methods/Design

### Objectives

The objective was to evaluate the efficacy of IMT associated with a CR program on modulating myocardial sympathetic activity and maximal functional capacity (primary outcome) as well as submaximal functional capacity (secondary outcome) and diaphragm muscle thickness and mobility (secondary outcome) in patients with HF.

### Study design

**Fig. 1:**
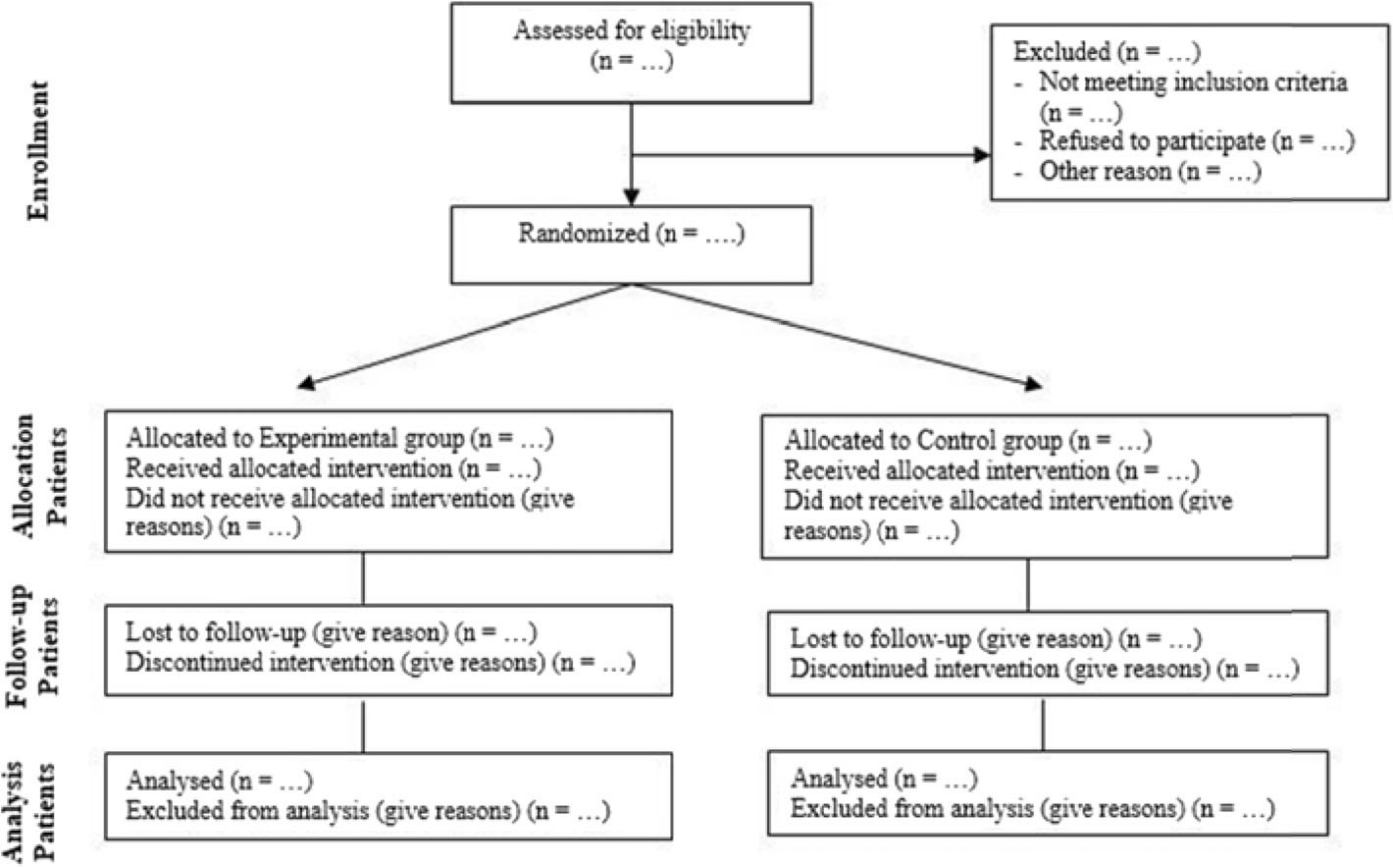
Study flowchart

This is a clinical, controlled, randomized, double-blind, and feasible trial developed in partnership with the Department of Physical Therapy of the Federal University of Pernambuco (*Universidade Federal de Pernambuco*) and *Hospital das Clínicas* of Pernambuco, and the patients come from the main reference centers of Recife-Brazil for the care of patients with HF. Figure 1 represents the follow-up flowchart of the study according to the CONSORT (Consolidated Standards of Reporting Trials) [12]. The sample will be calculated from the result of the first 20 patients through the GPower 3.1 software program [13] with α of 0.05 and a power of 80%. It will be calculated for all study variables, and the largest number will be increased by 20% to compensate for possible follow-up losses.

### Eligibility criteria

The patients included in the study are: adult sedentary men and women who are 21–60 years old, who have diagnosed systolic HF, a left ventricular ejection fraction of less than 45% evaluated by echocardiogram, functional class II and III (New York Heart Association), forced expiratory volume in the first second (FEV_1_) of less than 80% predicted and/or FEV_1_/ forced vital capacity (FVC) ratio of more than 70% predicted, inspiratory muscle weakness with a maximum inspiratory pressure (MIP) of less than 70% of predicted value for age and gender, and clinical stability and who are ex-smokers of more than 5 years and who have no change in the class of medications within 3 months prior to the start of the study. The patients will be excluded if presenting unstable angina; myocardial infarction or previous cardiac surgery up to 3 months before the beginning of the study; chronic respiratory diseases; smokers; hemodynamic instability; or recent face trauma, nausea, or vomiting (or a combination of these). Individuals with orthopedic and neurological diseases that may make it impossible to perform cardiopulmonary testing and CR exercises are also to be excluded, as are patients who present psychological alterations that restrict them from responding to the questionnaire.

### Intervention

Individuals will be allocated into one of two groups: the control group and the experimental group. The control group will be submitted to the conventional CR protocol, and the experimental group will follow the conventional CR protocol associated with IMT. The two proposed exercise protocols will have a frequency of three times a week for a period of 12 weeks.

#### Experimental group: conventional CR + IMT

The CR protocol consists of a supervised exercise program divided into two phases—aerobic exercise and strength training—with a goal of three sessions per week for a total of 36 sessions in 12 weeks. During the aerobic exercise phase, patients will initially walk on a treadmill or pedal on a stationary bicycle for 15 to 30 minutes per session continuously in a heart rate zone corresponding to the first ventilatory threshold obtained on the ergospirometric test. The exercise duration will be increased to 30 to 35 minutes after six sessions. The strength training phase will subsequently be performed with incremental load for upper and lower limbs.

The IMT will be conducted by the participants themselves seven times a week after being instructed how to do it by the lead researcher. These sessions will occur six times a week in the home environment and once a week at the research site, at which time there will be readjustment to the load. The IMT duration will also be 12 weeks. Powerbreathe® Classic – Light Resistance (Powerbreathe International Ltd., Southam, UK) equipment with a nasal clip at an intensity of 30% of the MIP will be used to perform the training [11] and will be evaluated through digital manovacuometry [14]. Powerbreathe® will be used offering a linear pressure load with a load range ranging from 10 to 90 cm H_2_O, with increments of 10 in 10 cm H_2_O with each turn given to the adjustment valve, so we will do a half turn on the valve when the determined load does not reach exact values, which allows an increase of 5 cm H_2_O.

The daily training will be 30 minutes stratified into three sets of 10 minutes or two sets of 15 minutes. Individuals will receive a training control card (training diary) to be filled in each training series and taken weekly to the researcher responsible for control. During training, patients will be instructed to inhale until the air enters and to maintain a respiratory rate between 12 and 16 incursions per minute. In order to minimize the information bias for both groups, we will also conduct telephone distance monitoring for all the days when we do not have personal contact with the patients.

#### Control group: conventional CR without IMT “Sham”

The CR protocol is the same for the experimental group. Sham is performed in a manner similar to that of the experimental group—daily training of 30 minutes stratified in series of 10 or 15 minutes—but the intensity does not exceed 10% of the MIP, which is not configured as training of the inspiratory musculature.

### Criteria for discontinuing or modifying the allocation

The criteria for discontinuing or modifying the allocation are worsening of symptoms, clinical instability, change in the class of medications, and emergence of serious infectious or viral diseases (or both) which are difficult to control.

### Strategies to improve adherence

The strategies to improve adherence are weekly educational lectures with topics related to the pathophysiological, nutritional, and medical aspects of HF and frequent connections to encourage rehabilitation and IMT daily as well as a training control diary that will be delivered to each participant.

### Evaluated outcomes

The participating individuals will be evaluated on the first day of evaluation by collecting their clinical history, applying the quality-of-life questionnaire, and reading the echocardiogram. Then they will be submitted to an anthropometric evaluation (height and weight through a digital scale with anthropometer [model W300, Welmy, São Paulo, Brazil] with a capacity of 300 kg, precision of 50 g, and an anthropometer with limit of 2 m and body mass index by dividing body weight by height squared), evaluation of vital signs through a multiparameter monitor (2023, Dixtal, Manaus, Brazil), evaluation of pulmonary function through spirometry, and evaluation of respiratory muscle strength through manovacuometry. Ultrasonography will be performed on the second day to measure the diaphragm muscle thickness and mobility (secondary outcome endpoint), and maximum functional capacity (primary outcome) will be evaluated by a responsible medical professional.

**Fig. 2:**
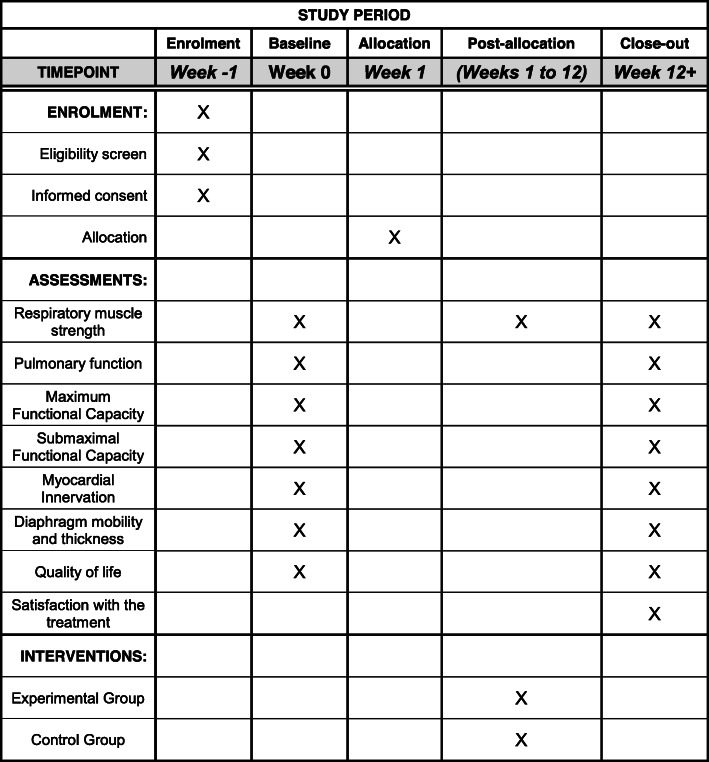
Standard protocol items: recommendations for interventional trials (SPIRIT) flowchart

The Glittre-ADL test will be performed on the third day for submaximal measurement of functional capacity (secondary outcome). Finally, the patients will be referred to a specialized hospital on the fourth day for myocardial scintigraphy (primary outcome). After all the evaluation procedures have been carried out and recorded on the corresponding file, the participants who are already randomly allocated will start their respective IMT protocols associated with CR; then the subjects will be submitted to the same evaluation process at the end of 36 sessions, along with a question that measures the degree of satisfaction with the treatment. Figure 2 and the checklist provide an overview of standardized items for clinical trials according to Standard protocol items: Recommendations for interventional trials (SPIRIT) [15].

### Randomization, allocation, and blinding

The subjects will be randomly assigned to groups through the website www.randomization.com in regard to the following protocols: control group (CR and “Sham” IMT) and experimental group (CR and IMT at 30% MIP). Then the allocation will be performed through numbered opaque envelopes by the researcher responsible for the weekly adjustment of the Powerbreathe load and they will only open the envelope corresponding to the patient at the time of the first adjustment. The entire randomization and allocation process will be carried out exclusively by the collaborating researcher, and the researcher will remain confidential until the end of the study. No other researcher or patients will be aware of which group they belong to, including the same one.

All of the patients will receive the training device and will have inspiratory muscle strength re-evaluated weekly to ensure patient masking, not being aware of whether the load is being increased or not. The device contains a protective cover that makes it impossible to visualize the imposed load. In addition, if the patient reports that the training is light, the researcher will tell them that it is natural and occurs as a result of training progression and improvement in muscle strength.

### Data collection, management, and analysis

#### Manovacuometry

An MVD-300 digital manovacuometer (Globalmed, Porto Alegre, Brazil) connected to a mouthpiece with a 2-mm orifice glottal closing pressure will be used. The volunteers will be placed seated, feet flat on the floor, erect spine, without supports for their upper limbs, using a mouthpiece and a nasal clip, and will be instructed to perform the MIP maneuver from the residual volume to total lung capacity (TLC), performing a maximum and sustained inspiration. There will be at least three maneuvers, a 1-minute interval between them, and reproducibility of 5–10% between maneuvers. The best among the three maneuvers will be adopted for recording the data. The predicted MIP is calculated for each individual through an equation for the Brazilian population [16].

#### Spirometry

Will be performed by using a portable spirometer (Micro Medical Microloop MK8, Micro Medical Ltd., Kent, UK). The volunteers will perform the maneuver seated, feet supported on the floor, spine erect, without support for their upper limbs and using a mouthpiece and nasal clip. At least three FVC maneuvers will be performed, and there will be a 2-minute interval between maneuvers in accordance with the reproducibility and acceptability criteria of the American Thoracic Society. Spirometric values are expressed as the percentage of the predicted normal value for the Brazilian population [17].

#### Ergospirometry

The symptom-limiting cardiopulmonary exercise test (CPET), considered the gold standard in the evaluation of functional capacity, will be performed for all patients in the study, using a ramp protocol [18] on a treadmill (Centurium 300, Micromed, Brasília, Brazil) through the ErgoPC Elite® software program associated with electrocardiogram (Micromed) with 12 channels. The respiratory variables will be evaluated by a gas analyzer (Metalyzer II, Cortex, Leipzig, Germany) obtained under standard conditions of temperature, pressure and dry (StPD), breath-by-breath, and the patient will be breathing in a non-leaking facial mask during exercise. Pressure, gas, and volume calibration of the equipment will be performed before each test. Examinations will be considered only where patients have a respiratory exchange ratio of at least 1.1 in order to ensure that patients achieve maximal effort during the examination. The test will be performed by an experienced cardiologist in an environment equipped with all emergency equipment and trained personnel. The test time is the time required to acquire a maximum stress test while maintaining patient safety since all vital signs and the electrocardiogram signal will be continually evaluated.

#### Myocardial scintigraphy with I^123^ MIBG

For myocardial scintigraphy with I^123^ MIBG, chest images will be obtained about 15 minutes after (early images) and 4 hours after (late images) receiving an intravenous injection of 185 MBq (5 mCi) of I^123^ MIBG. The images will be acquired in a tomographic gamma camera with a detector (Starcam model, General Electric Medical Systems, Milwaukee, WI, USA) with a collimator for low energy and high resolution. The energy photopeak will be centered on 159 KeV with a 20% window and 128 × 128 matrix. A static acquisition of 10 minutes will be performed in the anterior projection of the thorax in the early and late phases. A region of interest will be drawn manually over the heart (H) and over an area of nine pixels in the upper mediastinum (M) to obtain the mean counts of each of those regions, which forms the H/M ratio, it reflects the uptake of I MIBG in the nerve terminals. WR is expressed as a percentage and is calculated from the difference between early and late H/M ratios, indicating neuronal retention of the radiopharmaceutical in the area of the left ventricle. H/M values less than or equal to 1.8 and WR values greater than 19% will be considered abnormal (i.e., indicative of adrenergic hyperactivity). All patients will receive potassium iodide syrup (15 mL) orally about 1 hour before administration of I^123^ MIBG to block the thyroid gland [3]. Cardiac imaging with I^123^ MIBG is a non-invasive instrument for stratifying the risk of patients with HF by assessing sympathetic nervous system function in the heart muscle, providing treatment information and prognosis.

#### Glittre-ADL test

This test consists of carrying a backpack weighing 2.5 pounds for women and 5 kg for men, traversing a circuit with the following sequence of activities: getting up and walking out of the seated position, then walking a total distance of 10 m on the plane, interposed in its exact half (5 m) by a box with two steps to climb and two to descend, with a height of 17 cm and width of 27 cm of each step; going up and down the steps, and after traveling the rest of the course, moving three objects weighing 1 pound each placed on a shelf from the highest shelf to the lowest shelf, and then moving them to the floor; returning them to the middle shelf and higher shelf; and then returning through the entire course, sitting back in the chair and restarting another lap. The test consists of five rounds in total and individuals will be instructed to walk through them in the shortest possible time. No verbal stimulation will be given during the test. The participants will have their blood pressure, heart rate, and peripheral oxygen saturation measured and recorded before, during, and after the test through a tensiometer and stethoscope and portable pulse oximeter, respectively, in addition to perception of effort [19].

#### Ultrasonography of the diaphragm

A Sonoace R3 ultrasound (Samsung Medison, Pangyoyeok-ro, South Korea) will be used in B (thickness) and M (mobility) modes. In order to evaluate the diaphragm thickness, patients will be positioned in left lateral decubitus position and a linear low-penetration (7.5 MHz) high-resolution transducer will be positioned perpendicular to the thoracic cavity between the eighth and ninth intercostal space between the anterior and medial axillary line in such a way that it is possible to visualize two parallel bright lines that depict the pleural and peritoneal membrane. The thickness measurement will be carried out from the middle of the pleural line to the middle of the peritoneal line. An average of three final measurements of the thickness of the diaphragmatic apposition zone is performed, obtained during the functional residual capacity (FRC) (relaxed diaphragm thickness - Trel), at the end of the TLC (contracted diaphragm thickness - Tcont), and during a MIP maneuver from FRC; the latter will be used to determine the thickening rate (TR) of the diaphragm together with Trel, according to the formula:

$$ TR=\frac{Thickness\kern0.17em of\kern0.17em the\kern0.17em diaphragm\kern0.17em during\; MIP\; maneuver}{Average\kern0.17em thickness\kern0.17em of\kern0.17em the\kern0.17em diaphragm\kern0.17em in\kern0.17em the\; FRC} $$

In the diaphragm mobility evaluation, the patient will be positioned lying in the dorsal position with their thorax supported at a 45° inclination. A convex transducer (3.5 MHz) will be used in the right mid axillary line below the rib cage of the thoracic cavity, directed cranially. Patients will be instructed to breathe deeply and rapidly at the level of TLC and this maneuver will be repeated several times. The trajectory obtained between the baseline before the onset of inspiration and the plateau obtained at the end of the TLC will reveal diaphragm mobility, using the average of five measurements with a difference of less than 10% between them.

#### Quality-of-life evaluation

The Minnesota living with HF questionnaire, which is specific to the study population, will be used. It is composed of 21 items related to the causes of the problems occurring because of the heart problem. The last month should be considered to answer the questions. The scale of responses for each question varies from 0 (no) to 5 (others), where 0 represents without limitations and 5 represents maximum limitation. These issues involve a physical dimension (from 1 to 7, 12, and 13) which is highly interrelated with dyspnea and fatigue, an emotional dimension (from 17 to 21) and other issues (from numbers 8, 9, 10, 11, 14, 15, and 16), which form the total score when added to the previous dimensions [20].

#### Evaluation of the degree of satisfaction with the treatment

We have chosen to use the Patients Global Impression of Change (PGIC) scale in order to have access to interpretable information about the clinical importance of the changes in the health state perceived by the individuals when they undergo the intervention and their degree of satisfaction with it. The PGIC scale is a one-dimensional measure in which individuals rate their improvement associated with an intervention on a 7-item scale ranging from “1 = no change” to “7 = much better”. It was initially validated to determine clinically important minimal differences in degree of musculoskeletal pain [21, 22]. However, this instrument was adapted for use in our study as a way of evaluating the degree of patient satisfaction after the 3-month CR program.

### Statistical methods

Descriptive statistics will be used, and the continuous variables will be presented as means or medians, standard deviations, and 95% confidence intervals. Categorical variables will be expressed percentages and absolute numbers. The normality of the data will be evaluated through the Shapiro–Wilk or Kolmogorov–Smirnov tests. The Levene test will be used to test the variance homogeneity.

The paired *t* test will be used for normal data or the Wilcoxon test for non-normal data to compare the pre and post intervention of adrenergic tone, maximum and submaximal functional capacity, and diaphragm mobility and thickness. The unpaired *t* test or Mann–Whitney test will be used in the comparison between the groups depending on the normality of the data, and the significance level will be accepted at a *P* value of less than 0.05. The effect size will be calculated by using Cohen’s d, and the results will be interpreted on the basis of Cohen as follows: small (0.21–0.49), medium (0.50–0.79), or large (≥0.80) [23]. All participants included in the initial assessment will be considered under the intention-to-treat according to the method of last observation carried forward. The statistical analysis will be carried out with the Statistical Package for the Social Sciences, version 22.0 (SPSS Inc., Chicago, IL, USA).

### Ethical aspects

The research project was approved by the human research ethics committee of the Federal University of Pernambuco under protocol number CAAE: 38572614.1.0000.5208. It was registered on clinicaltrials.gov with the ID: NCT02600000.

After being recruited at the reference centers, all individuals in the target population will be clarified as to the research for which this proposal is available. To this end, each participant will sign the free and informed consent form after reading it aloud, in accordance with resolution number 466/12 of the National Health Council. Obtained information will be analyzed without disclosure of the data or identification of the individuals involved and will be used for study purposes and for completing the volunteer’s chart as a patient of the collaborating institutions. It will be clear that study participation will not affect the health care received by the participant in the service from which he or she was received. Data collection will be initiated only after the approval of this project by the referred commission.

Participating volunteers will be briefed on methodological steps and all evaluation processes. Each participant will be advised of the possibility to discontinue the process at any moment in which they choose and will not be charged with any onus in any manner. The possibility of adverse effects is minimal. Adverse phenomena such as nausea, dizziness, paleness, intense sweating, increased or decreased post-exercise pressure, increased or decreased heart rate per minute post-exercise, mild or moderate breathlessness, fatigue, or even cardiorespiratory arrest may occur.

However, in order to minimize any such effects, individuals will perform evaluation activities only when they are clinically stable. A suitable cardiologist for the ergospirometric test will be present and participate in the cardiopulmonary evaluation of all those involved. In addition, all professionals present will be able to attend emergency services for any possible intercurrences since they are all professionals with training in the health area and have knowledge in first aid, basic life support, and cardiopulmonary resuscitation. Moreover, there will be equipment and resources for pre-hospital care (oxygen cylinders, manual defibrillator, basic medical support supplies, and related drugs) in the laboratory in which the evaluations will be carried out, and it is also located close to one hospital which is an appropriate place for more complex approaches and which promises to receive patients in their emergency care unit in case of any adverse events. IMT does not present any risk for the patients who use it; proof of this is that it can be performed by the patient at home and without the need for supervision.

## Discussion

HF is a growing public health problem and has high morbidity and mortality rates [24]. This clinical trial is feasible and will be the first study to investigate the additional effects of IMT on CR, sympathetic activity, maximum and submaximal functional capacity, diaphragm mobility and thickness, and quality of life in patients with HF.

The IMT will be carried out through a validated instrument [25] and is suitable for this purpose. The device allows the training to be performed safely and unsupervised, but it has the limitation that it can be adjusted by only 10 cm H_2_O; thus, we will use approximate values in centimeters of water in an attempt to adjust this point. The results of this study will contribute to develop therapeutic strategies collaborating to elucidate whether the association of IMT with CR can induce clinical benefits for patients with HF.

## Trial status

This clinical trial has already been approved by the research ethics committee of the Federal University of Pernambuco, Recife, Brazil. It is currently in the recruitment and evaluation phase of the participants. The protocol of this research was outlined in January 2015, recruitment began in May 2015, and this phase is forecasted to close in December 2020.

## Data Availability

Not applicable.

## Abbreviations

ANS: Autonomic nervous system
CR: Cardiac rehabilitation
FEV_1_: Forced expiratory volume in the first second
FRC: Functional residual capacity
FVC: Forced vital capacity
HF: Heart failure
I^123^ MIBG: Metaiodobenzylguanidine labeled with iodine 123
IMT: Inspiratory muscular training
MIP: Maximum inspiratory pressure
PGIC: Patients Global Impression of Change
TLC: Total lung capacity
TR: Thickening rate
WR: Washout rate
